# Satisfaction with the walking-related environment during COVID-19 in South Korea

**DOI:** 10.1371/journal.pone.0266183

**Published:** 2022-04-20

**Authors:** Hoon Jo, Ho Hee Lee, Dong-Hyun Kim, In Deok Kong

**Affiliations:** 1 Artificial Intelligence Big Data Medical Center, Yonsei University Wonju College of Medicine, Wonju, South Korea; 2 Department of Rehabilitation Therapy, Hallym University Graduate School of Health Science, ChunCheon, South Korea; 3 Department of Preventive Medicine, Yonsei University Wonju College of Medicine, Wonju, South Korea; 4 Department of Social and Preventive Medicine, Hallym University College of Medicine; ChunCheon, South Korea; 5 Department of Physiology, Yonsei University Wonju College of Medicine, Wonju, South Korea; 6 Center for Exercise Medicine, Yonsei University Wonju College of Medicine, Wonju, South Korea; Shenzhen University, CHINA

## Abstract

This study aimed to examine the satisfaction level differences between urban and rural areas with regard to their walking environment during the COVID-19 pandemic in South Korea. This online cross-sectional research was conducted using a mobile health application. Overall, 1,032 local residents who participated in the mobile healthcare program of a public health center were classified as being from either urban (n = 481, 46.6%) or rural areas (n = 551, 53.4%) for the purpose of this study. The Walkability Checklist, which includes sociodemographic information, was employed using a Chi-square test and a multivariate logistic regression to investigate whether or not the participants were satisfied with the environmental factors associated with walking. It was found that both urban and rural areas were more likely to be unsatisfied with walking comfort (adjusted OR: 24.472, 95% CI: 14.937–40.096). Regarding the walking comfort aspects of the walking environment, urban residents chose poor landscape (“needed more grass, flowers, or trees”; aOR: 13.561, 95% CI: 3.619–50.823) as their primary dissatisfaction, and rural residents chose messy streets (“dirty, lots of litter or trash”; aOR: 29.045, 95% CI: 6.202–136.015). Compared with urban residents, rural residents were more discontented with the walking environment. Thus, to promote walking activities at the community level, it is necessary to focus on walking comfort, and implement efforts related to environmental beautification.

## Introduction

Insufficient physical activity is affecting the health of industrialized countries; furthermore, the burden of medical expenses is increasing [1]. Physical activities, such as flexibility, strength training, and walking, improve physical and mental health, reduce risk factors for chronic diseases, play an important role in body maintenance, and impact the quality of life of individuals [2]. Physical activities not only have a positive effect on disease prevention but are also highly related to national healthcare expenditures [3]. Physical activity is a significant strategy that can decrease the health problems of the elderly population, and regular practice can help them to be healthy, thus lessening the medical use rate and care cost [4, 5]. On March 11, 2020, the World Health Organization declared a global pandemic outbreak caused by the severe acute respiratory syndrome coronavirus (COVID-19) [6]. During the COVID-19 lockdown, restrictions on private gatherings, transportation, and industrial activities were put in place. Necessary methods such as social distancing and quarantine that limited physical activity and contact with numerous people, were implemented to prevent its spread [7]. It was under the influence of these limited activities that a reduced concentration of outdoor air pollutants was found [8–11]. Walking is a typical physical activity that can be performed in such a situation while maintaining social distance.

Walking is a low-intensity aerobic exercise with a low injury rate. It can be performed spontaneously anytime, anywhere, without any special equipment. Furthermore, since it has a low musculoskeletal burden, it can be applied to various age groups and is recommended for weight management purposes such as aerobic capacity [12], body composition, reduction of high blood pressure and blood lipid levels, and obesity [13]. Performing physical activities, such as walking for disease prevention, is important in chronic disease treatment that protects the immune system, and the facilitation of an appropriate response to the treatment of COVID-19 [14, 15].

To effectively increase the rates of physical activity and exercise, the implementation of an integrated strategy that comprises environmental change, policy modification, community-based programs, and media campaigns, is recommended [16]. In South Korea, efforts have been made to improve the physical activity and the walking practice rate of residents through health promotion programs. Core physical activity programs for the community include infrastructure building, supportive environment provision, community campaigns, health promotion education and public relations, physical activity programs for the elderly and obese, and exercise prescription programs [17]. Despite these efforts, the walking practice rate in Korea is only 42.6% for men and 37.1% for women, and demonstrates a downward trend [18]. During the COVID-19 pandemic, participation in walking activities has fluctuated according to the increase and decrease in COVID-19 cases, but it has not reached prior participation rates [19]. In this situation, factors related to walking activity promotion should be explored. However, detailed studies are lacking.

The Walkability Checklist collects subjective responses pertaining to various aspects of the walking environment in residents’ communities. These aspects include the creation of a walking space in the residential area, the pedestrian crossing environment, drivers’ consideration for pedestrians, and satisfaction with walking comfort [20]. Based on the responses of this tool, it is possible to identify areas of inadequacy in the community’s walking environment and to determine priorities regarding improvement measures. This study aimed to compare walking environment-related problems between urban and rural areas in South Korea, and find an environmental solution to promote walking activity.

## Materials and methods

### Study design and population

This cross-sectional research was conducted using an online survey. The participants lived in Gangwon-do, South Korea, and were registered applicants in a mobile healthcare program which was operated by public health centers. The mobile healthcare program is non-face-to-face and uses a mobile app and smart band. The purpose of this program is to prevent chronic diseases and improve the health management ability of participants by changing their health behavior. A program team composed of various field experts provided individual integrated health promotion services. All study participants were people who actively partook in walking activities around their residential areas. They used a health app called Walk-On (Swallaby Inc., Republic of Korea) and their participation was voluntary. The survey was initiated with the consent of the Gangwon Provincial Office and public health centers in Gangwon-do. Prior to the initiation of the study, the research participants consented to the provision and use of personal information and were provided with sufficient explanation about the research contents. Additionally, contents that could identify individuals were excluded from the survey questions. Those wishing to participate in the survey were asked to “start the survey” by entering their date of birth, thus confirming that they fully understand the contents of the survey and agree to participate. Overall, 1,032 individuals participated in the research, of which 481 (46.6%) and 551 (53.4%) lived in urban and rural areas, respectively. Their age ranged from 12–81 years, with a mean age of 50.0 ± 10.9 years.

### Data collection

Google Forms™ was used to create the survey and the questionnaires, including the ones for the investigation of satisfaction or dissatisfaction levels with regard to walking-related environmental factors. Google Forms is open to the public free of charge so that anyone can fill out a questionnaire and conduct a survey via a web link. A web link (https://forms.gle/WcL3AZxBaFmZTPSt6) containing the survey items was provided as a pop-up message in the Walk-On health app. This process was carried out with the app developer’s approval and the cooperation of the manager. The participants were able to access and complete the questionnaire from October 21 to December 20, 2020.

The questionnaire primarily consisted of the following: i) introduction of the questionnaire and information on consent to participation, ii) Walkability Checklist [20], and iii) general information collection items including residential area (S1 and S2 Files). The first part of the survey provided the participants with information about the main objectives of this study. Additionally, their consent regarding anonymity and voluntary participation was obtained. To ensure the former, no personal identifiable information was collected. The submission of the completed questionnaire implied that the participants consented to take part in the research. Incomplete response data were excluded from the subsequent analysis. The items in the Walkability Checklist were partially modified to fit the purpose of this study. The response to the questions were modified based on a five-point Likert scale, and the fourth question, “Was it easy to follow safety rules?,” was excluded from the questionnaire.

### Sociodemographics and other variables

Sociodemographic data such as gender, age, and marital status were collected to determine the participants’ characteristics. Information on their residence period and the residential type were obtained to evaluate the state of the physical walking environment at the individual level. Moreover, data on employment status and participation in walking clubs were investigated to discover the relationship of social status with the walking environment.

Age and residence period were divided into five (≤ 30, 31~40, 41~50, 51~60, ≥ 61 years) and three categories (≤ 5, 5~20, ≥ 20 years), respectively. Marital status was divided into two categories: “married” and “single, divorced, widowed,” and residential types were divided into three categories (“detached house,” “multi-family house,” and “apartment”). Employment status was identified as “employed,” “not employed,” and “other,” and participation in walking clubs was additionally investigated. All sociodemographic information was answered by the survey participants directly on the online app.

### Satisfaction or dissatisfaction with the walking-related environmental factors

Walkability is affected by various factors such as whether or not a walking area has been established, the stability of the walking activity, traffic behavior, and walking comfort. Accordingly, we selected the four related items from the Walkability Checklist [13] and used them as the primary questions (“Did you have space to walk?,” “Was it easy to cross the streets?,” “Did the drivers behave appropriately?,” and “Was your walk pleasant?”). The responses for these four major factors were collected on the basis of a five-point Likert scale (where 1, 2, 3, 4, and 5 represented absolutely not, no, neutral, yes, and very much, respectively). To divide these answers into satisfaction or dissatisfaction, we recategorized 1, 2, and 3 as dissatisfied and 4 and 5 as satisfied. If the participant’s response was that of dissatisfaction in "Was your walk pleasant?", duplication of the response may be allowed as the cause of dissatisfaction. The responses to six representative problems (“needed more grass, flowers, or trees,” “not well-lit,” “dirty, lots of litter or trash,” “unclean air due to automobile exhausts,” “scary people,” and “frightening dogs or other threats”) were extracted from the walking comfort-related issues in the Walkability Checklist. Although the Walkability Checklist is limited by its low predictive validity, it is possible to identify problems between various components through the evaluation of actual users [21].

### Statistical analysis

A Chi-squared test evaluated associations regarding walking-related environmental factors between the urban and rural areas. The outcome is a dichotomous variable of satisfaction or dissatisfaction based on the responses of the four main questions. A logistic regression analysis was conducted to determine the odds ratio of dissatisfaction for the four major walking-related environmental factors (“condition of the walking space,” “pedestrian crossing environment,” “drivers’ consideration for pedestrians,” and “comfort of the walking activity”). Additionally, after stratification into urban and rural areas, the most influential factor was identified by comparing the odds ratio. It was performed using two models: the crude and the adjusted. The latter was performed using a multiple logistic regression analysis, that accounted for gender, age, marital status, region, residential type, residence period, and employment status. It was examined with the Cox and Snell’s coefficient of determination. The multicollinearity among the variables in this model was assessed using the variance inflation factor. All statistical analyses were performed with the R statistical package version 4.0.5 [22]. The statistical tests were two-sided, and the statistical significance was set at p < 0.05.

### Ethical considerations

The study protocol was reviewed and approved by the Institutional Review Board (IRB) of the Wonju Severance Christian Hospital (CR321306), Wonju, South Korea.

## Results

### General characteristics of the participants

Table 1 presents the participants’ sociodemographic information and their involvement in the walking clubs. The proportion of women was relatively higher (n = 694, 67.2%), and over half of the participants (n = 690, 66.9%) were in their 40s (n = 336, 32.6%) and 50s (n = 354, 34.3%). The majority of participants’ residence period was between 6–19 years (n = 429, 41.6%), followed by more than 20 years (n = 289, 28.0%), which accounted for more than two-thirds of the sample. Over half of the participants lived in apartments (n = 556, 53.9%) and were employed (n = 587, 56.9%). Only about one in five answered “yes” to walking club participation (n = 208, 20.2%).

**Table 1:** Sociodemographic characteristics of the participants.

	Overall (n = 1032)		Urban n = 481		Rural n = 551		P-value
	*n*	*%*	*n*	*%*	*n*	*%*	
***Gender***							
Male	338	32.8	186	38.7	152	27.6	< .001
Female	694	67.2	295	61.3	399	72.4	
***Age (in years)***							
≤ 30	52	5.0	28	5.8	24	4.4	.024
31~40	136	13.2	74	15.4	62	11.3	
41~50	336	32.6	167	34.7	169	30.6	
51~60	354	34.3	152	31.6	202	36.6	
≥ 61	154	14.9	60	12.5	94	17.1	
***Marital status***							
Married	817	79.2	365	75.9	452	82.0	.015
Unmarried/Divorced/Widowed	215	20.8	116	24.1	99	18.0	
***Residence Period (in years)***							
≤ 5	314	30.4	175	36.4	139	25.2	< .001
6~19	429	41.6	207	43.0	222	40.3	
≥ 20	289	28.0	99	20.6	190	34.5	
***Residential type***							
Single-family house	357	34.6	67	13.9	290	52.6	< .001
Row-house	119	11.5	47	9.8	72	13.1	
Apartment	556	53.9	367	76.3	189	34.3	
***Employment status***							
Employed	587	56.9	280	58.2	307	55.7	.297
Unemployed	217	21.0	91	18.9	126	22.9	
Others	228	22.1	110	22.9	118	21.4	
***Participation in walking clubs***							
Yes	208	20.2	66	13.7	142	25.8	< .001
No	824	79.8	415	86.3	409	74.2	

Regarding the difference in the distribution between urban and rural areas, the ratio of women in the latter (n = 399, 72.4%) was higher than that in the former (n = 295, 61.3%); moreover, this difference was statistically significant (p < .001). Additionally, about half of the rural ones reported residing in single-family houses (n = 290, 52.6%) compared with over two-thirds of the urban residents who were living in apartments (n = 367, 76.3%), which was statistically significant (p < .001).

### Differences in the satisfaction of walking-related environmental factors according to residential area

Table 2 shows the results of the comparison analysis for the satisfaction of walking-related environmental factors based on residential area. Compared with urban residents, rural residents had a relatively high proportion of dissatisfaction for all walking-related environmental aspects investigated. Further, 286 (59.5%) and 349 people (63.3%) in the urban and rural areas, respectively, reported dissatisfaction with “drivers’ consideration for pedestrians.” Although there was no statistically significant difference between the two groups, over half of them were discontented. In contrast to 29.7% of the urban residents who were dissatisfied with the condition of the walking space, 42.5% of the rural ones reported the same, which was statistically significant (χ^2^ = 17.973, p<0.001). Regarding pedestrian consideration, 32% and 45.9% of the urban and rural area inhabitants, respectively, demonstrated being disappointed; this result was statistically significant (χ^2^ = 20.774, p<0.001). Additionally, the dissatisfaction rate with respect to walking activity comfort among the latter was also significantly higher (χ^2^ = 8.882, p<0.01).

**Table 2:** Differences in the satisfaction of the walking-related environmental factors according to the residential area.

	Urban (n = 481)	Rural (n = 551)	χ^2^	P-value
***Condition of the walking space***				
Satisfied	338 (70.3)	317 (57.5)	17.973	< .001
Dissatisfied	143 (29.7)	234 (42.5)		
***Pedestrian crossing environment***				
Satisfied	327 (68.0)	298 (54.1)	20.774	< .001
Dissatisfied	154 (32.0)	253 (45.9)		
***Drivers’ consideration for pedestrians***				
Satisfied	195 (40.5)	202 (36.7)	1.633	.201
Dissatisfied	286 (59.5)	349 (63.3)		
***Comfort of the walking activity***				
Satisfied	331 (68.8)	330 (59.9)	8.882	.003
Dissatisfied	150 (31.2)	221 (40.1)		

Table 3 presents the logistic regression analysis results of the odds ratio of dissatisfaction with the walking-related environment factors. This odds ratio and that of dissatisfaction with the “comfort of the walking activity” in the area of residence was 21.538 (OR: 21.538, 95% CI: 13.491–34.386); furthermore, when the associated variables were adjusted, it was 24.472 times higher (aOR: 24.472, 95% CI: 14.937–40.096). This trend was higher in rural areas than in urban areas; moreover, when the residents of the former were dissatisfied with the walking comfort, the likelihood of being discontented with the overall walking environment increased significantly (OR: 27.727, 95% CI: 13.939–55.157; aOR: 34.360, 95% CI: 16.208–72.840).

**Table 3:** Odds ratios of dissatisfaction with the walking-related environments.

	Overall		Urban		Rural	
	*Crude OR (95% CI)*	*Adjusted OR*^*a*^ *(95% CI)*	*Crude OR (95% CI)*	*Adjusted OR*^*b*^ *(95% CI)*	*Crude OR (95% CI)*	*Adjusted OR*^*b*^ *(95% CI)*
***Dissatisfied with***						
Condition of the walking space	9.601 (6.049–15.241)	10.760 (6.593–17.561)	9.308 (4.755–18.220)	11.655 (5.564–24.414)	9.734 (5.096–18.594)	12.600 (6.165–25.752)
Pedestrian crossing environment	3.546 (2.255–5.576)	3.735 (2.308–6.045)	2.325 (1.203–4.496)	2.544 (1.255–5.158)	5.459 (2.847–10.465)	5.326 (2.612–10.862)
Drivers’ consideration for pedestrians	2.662 (1.660–4.269)	2.875 (1.760–4.696)	3.417 (1.741–6.707)	3.699 (1.818–7.526)	2.054 (1.045–4.034)	2.542 (1.230–5.255)
Comfort of the walking activity	21.538 (13.491–34.386)	24.472 (14.937–40.096)	17.926 (9.311–34.510)	22.118 (10.850–45.087)	27.727 (13.939–55.157)	34.360 (16.208–72.840)

Abbreviations: OR, odds ratio; CI, confidence interval.

^a^adjustments: gender, age, marital status, region, residential type, residence period, job.

^b^adjustments: gender, age, marital status, residential type, residence period, job.

### Odds ratios for the dissatisfaction with walking-related environments according to factors associated with walking activity comfort

**Fig 1:**
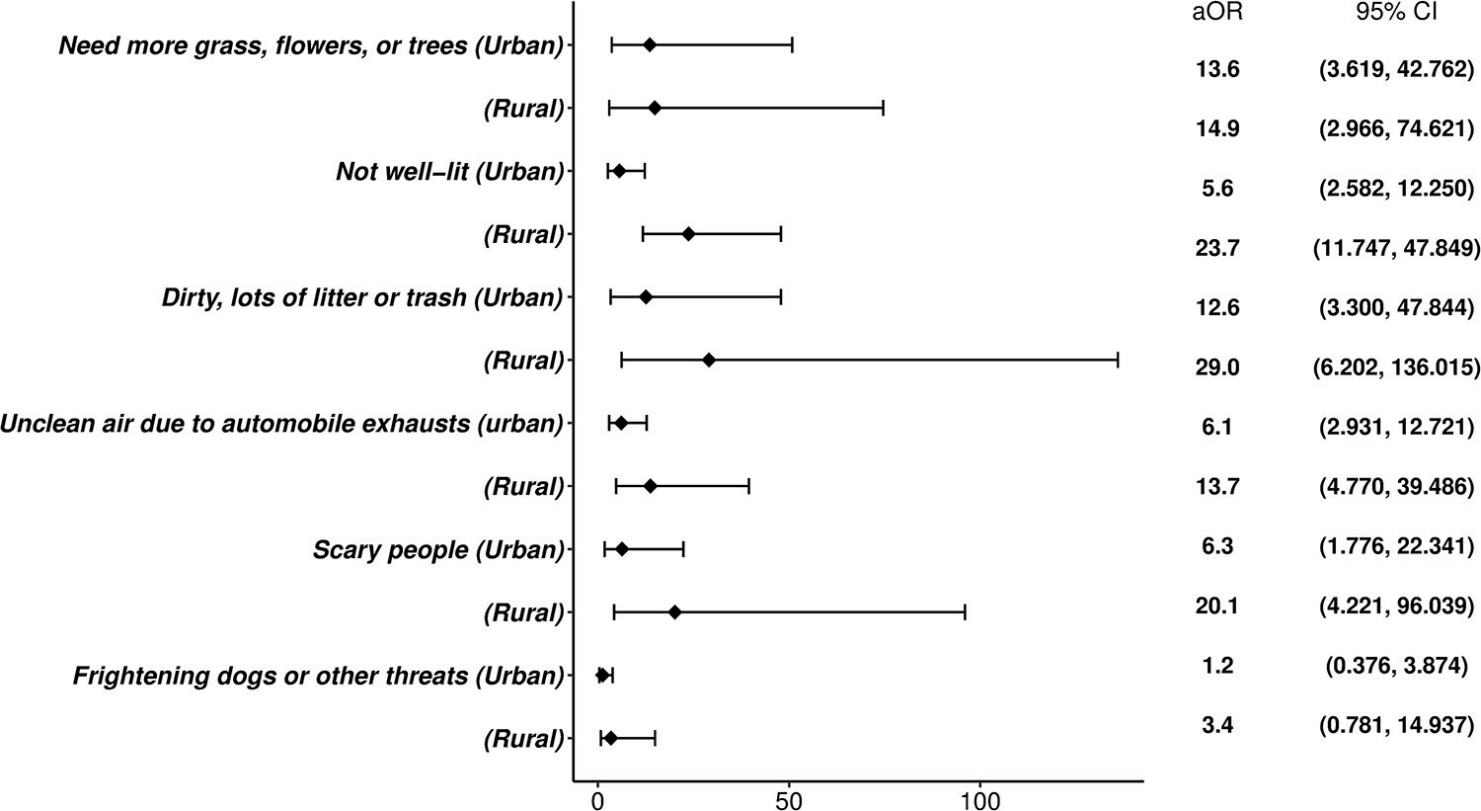
Comparisons with adjusted odds ratios of walking activity comfort factors. aOR, adjusted odds ratio; CI, confidential interval.

Table 4 displays the odds ratio of the dissatisfaction with the walking environment based on the factors related to the walking activity comfort. Overall, both urban and rural areas were most likely to be dissatisfied with “dirty, lots of litter or trash” (OR: 17.043, 95% CI: 6.414–45.285; aOR: 17.588, 95% CI: 6.505–47.552). Even when the analysis was limited to the latter, displeasure with this aspect demonstrated the highest possibility of disappointment with the overall walking environment (OR: 25.629, 95% CI: 5.733–114.573; aOR: 29.045, 95% CI: 6.202–136.015). The factors with the subsequent highest odds ratio were “not well-lit” and “scary people.” However, in the urban areas, the odds ratio for “needed more grass, flowers, or trees” was the highest when the associated variables were adjusted (aOR: 13.561, 95% CI: 3.619–50.823), and the ratio for “dirty, lots of litter or trash” (aOR: 12.566, 95% CI: 3.300–47.844) followed (Fig 1).

**Table 4:** Odds ratios for the dissatisfaction with the walking-related environments according to the factors associated with the walking activity comfort.

	Overall		Urban		Rural	
	*Crude OR (95% CI)*	*Adjusted OR*^*a*^ *(95% CI)*	*Crude OR (95% CI)*	*Adjusted OR*^*b*^ *(95% CI)*	*Crude OR (95% CI)*	*Adjusted OR*^*b*^ *(95% CI)*
***Factors related to the walking activity comfort***						
Needed more grass, flowers, or trees	12.630 (4.663–34.207)	13.733 (4.981–37.862)	11.760 (3.234–42.762)	13.561 (3.619–50.823)	15.063 (3.194–71.037)	14.877 (2.966–74.621)
Not well-lit	11.379 (7.039–18.394)	12.129 (7.391–19.906)	5.401 (2.568–11.359)	5.624 (2.582–12.250)	19.984 (10.278–38.857)	23.708 (11.747–47.849)
Dirty, lots of litter or trash	17.043 (6.414–45.285)	17.588 (6.505–47.552)	12.033 (3.275–44.204)	12.566 (3.300–47.844)	25.629 (5.733–114.573)	29.045 (6.202–136.015)
Unclean air due to automobile exhausts	7.577 (4.248–13.516)	7.657 (4.219–13.897)	5.725 (2.823–11.608)	6.107 (2.931–12.721)	13.310 (4.792–36.967)	13.725 (4.770–39.486)
Scary people	10.258 (4.088–25.742)	10.621 (4.118–27.393)	5.769 (1.729–19.249)	6.299 (1.776–22.341)	20.956 (4.646–94.522)	20.134 (4.221–96.039)
Frightening dogs or other threats	1.630 (0.645–4.123)	1.572 (0.616–4.015)	1.305 (0.408–4.171)	1.207 (0.376–3.874)	3.358 (0.827–13.626)	3.415 (0.781–14.937)

Abbreviations: OR, odds ratio; CI, confidence interval.

^a^adjustments: gender, age, marital status, region, residential type, residence period, job.

^b^adjustments: gender, age, marital status, residential type, residence period, job.

## Discussion

This study aimed to present an effective environmental improvement plan to promote walking activities that are relatively unrestricted by time and space during the COVID-19 pandemic. Using a structured checklist, satisfaction levels related to various aspects associated to the walking environment were identified, and their relative proportion to the factors of dissatisfaction was confirmed. Consequently, the research confirmed that compared to urban areas, rural areas were more likely to be discontented with environmental factors related to walking activities. Regarding walking comfort, “dirty, lots of litter or trash” was a source of displeasure that had the most influence on the overall dissatisfaction with the walking environment.

Regular physical activity must be encouraged for people to help them maintain a healthy immune system and bodily functions during unstable circumstances such as the COVID-19 pandemic [23]. From this perspective, walking exercises that can be performed while maintaining social distance should be further recommended, and various interventions to promote them should be implemented. Therefore, it is necessary to select an effective strategic plan despite limited financial resources and find the most effective method. The emergence of the COVID-19 pandemic has resulted in positive environmental benefits due to restrictions on various industrial activities and transportation [9]. The reduction of environmental pollutants prompted a more pleasant outdoor environment [8, 10, 11]. In these circumstances, maintaining social distance and actively performing physical activity can provide reasonable health benefits. Health authorities need to encourage physical activities like walking, and monitor changes in the levels of environmental pollution.

This study found that the proportion of dissatisfaction with all walking-related environmental factors was higher in rural residents. People living in rural regions are generally older and have lower income and education levels than those in urban areas, which can lead to differences in their health-related physical activity performance [24]. Therefore, it becomes more challenging for the former to perform physical activities, including walking, because their walking environment is poorly established. These residents can be physically active when a safe and accessible walking environment is created around their residential area [25]. Therefore, for rural areas, strategies to enhance walking conditions, secure pedestrian stability, and provide walking comfort that centers on residential areas, should be proposed to reduce the gap with urban areas. For both urban and rural regions, over half of the respondents reported their dissatisfaction with “driver’s consideration for pedestrians,” which thus confirmed the need for education regarding drivers’ concern toward walkers, and an increase of fines.

Several studies have found that pedestrians generally walk farther for recreation rather than for travel among U.S. residents [26, 27]. Specifically, people with high income levels and those living outside the city tended to walk relatively long distances for recreational purposes [27]. A study conducted in Minneapolis–Saint Paul showed that people walk longer distances for recreational and exercise purposes than for other purposes [28]. From these findings, it can be inferred that the promotion of walking activity should focus on the provision of sufficient recreation rather than the simple replacement of the means of transportation for the purpose of moving. Thus, providing a comfortable walking environment is an effective way to increase pedestrian satisfaction. Environmental factors directly impact the likelihood of the performance of physical activities [29, 30]. Specifically, “dirty, lots of litter or trash” was the greatest element of dissatisfaction with the walking environment. It is necessary to prioritize the improvement of the pedestrian environment and keep the pedestrian path clean. In the case of urban areas, it showed a high correlation with “needed more grass, flowers, or trees”; hence, in these regions, environmental beautification and financial investment to enhance aesthetics will provide motivation for the conduction of walking activities. Additionally, the absence of walking companions lowers participation in such exercises [31]. Thus, social environment support, such as a walking club, should be provided.

This study has several limitations. As the survey only included participants partaking in the mHealth care program, it cannot represent the entire local population. However, it can be considered that reasonable information on the walking environment was provided because the respondents actively performed walking activities around their residential area. Additionally, the validity may have been reduced because the survey was conducted in a self-report format rather than as an objective measurement.

Nevertheless, this study presents several key points compared with those of previous studies. First, it was conducted when the COVID-19 pandemic had intensified, and reflected changes in the previous perceptions of and participation in walking exercises. Second, specific problems related to walking activities in urban and rural areas in South Korea were derived and compared, instead of the use of a checklist that is applicable worldwide. Finally, in the context of the COVID-19 pandemic, the study is meaningful in that it suggests an effective solution—walking activities to promote the health of local residents living in urban and rural areas.

## Conclusion

Promotion of physical activities, such as walking exercises, is essential not only during the COVID-19 pandemic, but also to lead a normal healthy life. Most of the local residents showed that the satisfaction level of the walking environment changed according to the level of walking comfort; further, it was confirmed that the maintenance of a clean environment can enhance walking activity. Additionally, in urban areas, walking environment beautification may be suggested to increase satisfaction. It is expected that health promotion will be achieved by identifying the needs of local residents and implementing effective intervention strategies.

## Supporting information

S1 File A questionnaire of Walkability Checklist and general information of respondents (English). (PDF)

S2 File A questionnaire of Walkability Checklist and general information of respondents (Korean). (PDF)
