# Supplementary material for: Satisfaction with the walking-related environment during COVID-19 in South Korea
Source: PLoS One. 2022 Apr 20;17(4):e0266183. doi: 10.1371/journal.pone.0266183 (PMC9020742; doi:10.1371/journal.pone.0266183)
Supplement: S1 File — (PDF) [file pone.0266183.s001.pdf]

1. Walkability Checklist

Q1. Did you have room to walk?

①	②	③	④	⑤
Strongly disagree	Disagree	Neutral	Agree	Strongly agree

Q1-1. **[Only the respondents who chose ①~③ in Q1]** Why are you dissatisfied with the above question? **(Please check all that apply)**

1) Sidewalks or paths started and stopped
2) Sidewalks were broken or cracked
3) Sidewalks were blocked with poles, signs, shrubbery, dumpsters, etc.
4) No sidewalks, paths, or shoulders
5) Too much traffic
6) Something else (   )

Q2. Was it easy to cross streets?

①	②	③	④	⑤
Strongly disagree	Disagree	Neutral	Agree	Strongly agree

Q2-1. **[Only the respondents who chose ①~③ in Q2]** Why are you dissatisfied with the above question? **(Please check all that apply)**

1) Road was too wide to cross

2) Traffic signals made us wait too long

3) Traffic signals did not give us enough time to cross

4) Needed striped crosswalks or traffic signals

5) Parked cars blocked our view of traffic

6) Needed curb ramps or ramps needed repair

7) Dead end road that's not connected to the destination

8) Something else (   )

Q3. Did drivers behave well?

①	②	③	④	⑤
Strongly disagree	Disagree	Neutral	Agree	Strongly agree

Q3-1. **[Only the respondents who chose ①~③ in Q3]** Why are you dissatisfied with the above question?

(Please check all that apply)

1) Backed out of driveways without looking

2) Did not yield to people crossing the street

3) Turned into people crossing the street

4) Drove too fast

5) Sped up to make it through traffic lights or drove through traffic lights?

6) Something else (   )

Q4. Was your walk pleasant?

①	②	③	④	⑤
Strongly disagree	Disagree	Neutral	Agree	Strongly agree

Q4-1. **[Only the respondents who chose ①~③ in Q4**] Why are you dissatisfied with the above question?

(Please check all that apply)

1) Needed more grass, flowers, or trees
2) Not well lighted
3) Dirty, lots of litter or trash
4) Dirty air due to automobile exhaust
5) Scary people
6) Scary dogs
7) Something else ( )

2. General information of respondents

1. Gender	① Male ② Female
2. Age	________
3. Location	Gangwon-do Province<br>1) Gangneung-si 2) Goseong-gun 3) Donghae-si<br>4) Samcheok-si 5) Sokcho-si 6) Yanggu-gun<br>7) Yangyang-gun 8) Yeongwol-gun 9) Wonju-si<br>10) Inje-gun 11) Jeongseon-gun 12) Cheorwon-gun<br>13) Chuncheon-si 14) Taebaek-si 15) Pyeongchang-gun<br>16) Hongcheon-gun 17) Hwacheon-gun 18) Hoengseong-gun
4. Residential type	① single-family house<br>② row house/Multi-family house, Multiplex House<br>③ Apartment<br>④ Officetel (dual-purpose buildings used for commercial and residential purposes)<br>⑤ Etc. ( )
5. Residence Period	① Under 3 years<br>② 3-4 years<br>③ 5-9 years<br>④ 10-19 years<br>⑤ Over 20 years
6. Employment Status	① Office job (salaried worker etc.)<br>② specialized job (doctor, lawyer, professor, accountant etc.)<br>③ civil servant<br>④ Production job/General Labor<br>⑤ Primary industry worker (agriculture, forestry, livestock industry etc.)<br>⑥ Self-employed<br>⑦ sales job/service job

	⑧ A homemaker<br>⑨ Military<br>⑩ A student/repeater<br>⑪ Unable to work<br>⑫ Etc. ( )
7. Marital Status	① Married (Living together) ② Single, never married<br>③ Etc. (divorced, Widowed, Separated...)
8. Family members (including yourself)	**Total _____**<br>**※ Please select all family members currently living with you.**<br>① You (including singles)<br>② Spouse<br>③ Children ( )<br>④ Parents<br>⑤ Siblings<br>⑥ Etc. ( )
9. Participation in walking clubs	Do you participate in regular walking club activities?<br>① Yes<br>② No<br><br>Q. 12-1.[**Only the respondents who chose ① in above question]** The name of the walking club you are currently participating in**: ( )**
10. Pet dog	Do you currently have a dog? ① Yes ② No
Phone number	
