# Supplementary material for: Satisfaction with the walking-related environment during COVID-19 in South Korea
Source: PLoS One. 2022 Apr 20;17(4):e0266183. doi: 10.1371/journal.pone.0266183 (PMC9020742; doi:10.1371/journal.pone.0266183)
Supplement: S2 File — (PDF) [file pone.0266183.s002.pdf]

1. 도보환경평가

Q1. 귀하께서 거주하고 계신 지역의 도보(걷기) 공간이 잘 조성되어 있습니까?

①	②	③	④	⑤
전혀 그렇지 않다	그렇지 않다	보통	그렇다	매우 그렇다

Q1-1. [Q1에서 ***①~③ 응답자만***] 위 문항에 대한 불만족 이유는 무엇입니까? **(중복응답)**

① 보도(인도)가 중간에 끊어짐

② 보도(인도)가 파손 또는 균열됨

③ 보도(인도)가기둥,간판,쓰레기통등으로막힘

④ 보도(인도) 또는 갓길이 부족함

⑤ 교통량(차량)이 너무 많음

⑥ 기타 (   )

Q2. 귀하께서는 거주하고 계신 지역의 길 건너기 환경은 잘 조성되어 있습니까?

①	②	③	④	⑤
전혀 그렇지 않다	그렇지 않다	보통	그렇다	매우 그렇다

Q2-1. [Q2에서 ***①~③ 응답자만***] 위 문항에 대한 불만족 이유는 무엇입니까? **(중복응답)**

① 길이 너무 넓어서 건너기 어려움

② 신호가 길어서 오래 기다려야 함

③ 길 건너기에는 신호시간이 짧음

④ 횡단보도 또는 교통신호가 부족함

⑤ 주차차량 등이 교통시야를 가림

⑥ 도로 경계석이 없거나 위험한 지면

⑦ 목적지까지 연결되지 않은 막다른 길

⑧ 기타 (   )

Q3. 귀하께서 거주하고 계신 지역의 경우, 운전자들이 보행자를 배려한다고 생각하십니까?

①	②	③	④	⑤
전혀 그렇지 않다	그렇지 않다	보통	그렇다	매우 그렇다

Q3-1. **[**Q3에서 ***①~③ 응답자만*]** 위 문항에 대한 불만족 이유는 무엇입니까? **(중복응답)**

① 뒤를 살펴보지 않고 진입로에서 후진

② 길 건너는 사람에게 양보하지 않음

③ 보행자를 잘 보지 않고 운행함

④ 과속함

⑤ 정지신호 또는 신호등을 무시함

⑥ 기타 (   )

Q4. 귀하께서 거주하고 계신 지역의 경우, 걷기에 쾌적하다고 생각하십니까?

①	②	③	④	⑤
전혀 그렇지 않다	그렇지 않다	보통	그렇다	매우 그렇다

Q4-1. **[**Q4에서 ***①~③ 응답자만*]** 위 문항에 대한 불만족 이유는 무엇입니까? **(중복응답)**

① 자연환경이 너무 삭막함 (거리에 가로수, 꽃 등이 부족함)

② 조명이 어두움

③ 거리가 청결하지 않고 공터가 지저분함

④ 자동차 배기로 인한 공기오염

⑤ 범죄 또는 안전사고에 대한 두려움

⑥ 유기견 또는 그 밖의 위협

⑦ 기타 (   )

2. 응답자 일반사항

1. 성	①남성　　②여성
2 연령	만 __ __세
3. 거주지역	강원도<br>1)강릉시 2)고성군 3)동해시 4)삼척시 5)속초시 6)양구군<br>7)양양군 8)영월군 9)원주시 10)인제군 11)정선군 12)철원군<br>13)춘천시 14)태백시 15)평창군 16)홍천군 17)화천군 18)횡성군
4. 주택 유형	①단독주택<br>②연립/다세대/다가구주택, 빌라<br>③아파트<br>④오피스텔<br>⑤기타(　　　　　　)
5. 거주기간	①3년 미만<br>②3~5년 미만<br>③5~10년 미만<br>④10년~20년 미만<br>⑤20년 이상
6. 직업	①사무직(일반회사원 등)<br>②전문직(의사, 변호사, 교수, 회계사 등)<br>③공무원<br>④생산직/노무직<br>⑤1차산업종사자(농업, 임업, 축산업 등)

	⑥자영업<br><br>⑦판매직/서비스직<br><br>⑧전업주부<br><br>⑨군인<br><br>⑩학생/재수생<br><br>⑪무직<br><br>⑫기타( )
7. 혼인상태	①배우자 있음(같이 살고 있음)<br><br>②미혼<br><br>③기타(이혼, 사별, 별거 등)
8. 가족구성원<br><br>(본인 포함)	**총 ___명**<br>**※현재 함께 거주하고 있는 가족 구성원을 모두 선택해 주세요.**<br>①본인(독신 포함)<br><br>②배우자<br><br>③자녀( )명<br><br>④부모<br><br>⑤형제자매<br><br>⑥기타( )
9. 걷기동호회<br><br>참여 여부	선생님은 정기적인 걷기동호회 활동에 참여하고 계십니까?<br><br>①예<br><br>②아니오

	12-1. [***"① 예" 응답자만***] 현재 참여중인 걷기동호회 이름: ( )
10. 반려견 유무	선생님은 현재 반려견을 키우고 계십니까? ①예 ②아니오
전화번호	
